# LGBT WIDOWHOOD: THE ASSOCIATION BETWEEN PARTNER LOSS AND PSYCHOLOGICAL WELL-BEING

**DOI:** 10.1093/geroni/igz038.1108

**Published:** 2019-11-08

**Authors:** Jessica A Noblitt, Anne Barrett

**Affiliations:** Florida State University, Tallahassee, Florida, United States

## Abstract

Death of a partner – one of the most stressful events many people will ever experience – has profound effects on psychological well-being. However, research on widowhood focuses almost exclusively on heterosexual couples, with little known about these studies’ applicability to the LGBT population. Further, the few studies of partner loss among LGBT individuals are qualitative and many focus on gay men. As a result, little is known about the effects of partner loss in this population as a whole or among other sexual minorities, including lesbians, bisexuals, and transgender individuals. Addressing this issue, our study examines the association between partner loss and psychological well-being using data from the 2010 Aging with Pride: National Health, Aging, and Sexuality/Gender Study (NHAS) (n=2,322), the largest nationally representative sample of LGBT middle-aged and older adults. We use OLS regression to examine the association between partner loss and psychological well-being, measured as depressive symptoms, loneliness, and subjective mental health. Results reveal a significant association between partner loss and psychological well-being across all three measures. However, the association is significantly weaker in models controlling for current partner status. In models predicting depressive symptoms and loneliness, partner loss no longer reaches significance, though it remains significant in the model predicting subjective mental health. Results suggest that current partner status more strongly impacts psychological well-being than does partner loss, though further research is needed to identify how, for whom, and under what circumstances the negative effects of partner loss may linger.

